# Supplementary material for: Causal Relationship between Aging and Anorexia Nervosa: A White-Matter-Microstructure-Mediated Mendelian Randomization Analysis
Source: Biomedicines. 2024 Aug 16;12(8):1874. doi: 10.3390/biomedicines12081874 (PMC11351342; doi:10.3390/biomedicines12081874)
Supplement: Supplementary file 1 [file biomedicines-12-01874-s001.zip › Supplementary Figure/Figure S8/Leaveoneout plot of MR analysis for TL to ubm-b-1748.pdf]

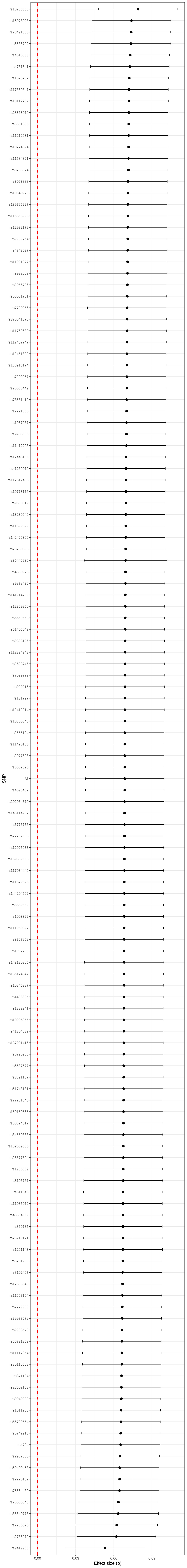
rs10768683
rs16978028
rs78491606
rs6536702
rs4616688
rs4731541
rs1023767
rs117630647
rs10112752
rs28363070
rs6881568
rs11212631
rs10774624
rs11584821
rs3785074
rs3093888
rs10840270
rs139795227
rs116863223
rs12932179
rs2282764
rs4743037
rs11991877
rs932002
rs2056726
rs56061761
rs7790856
rs376641875
rs11769630
rs117407747
rs12451892
rs188918174
rs7209057
rs76666449
rs73581419
rs7221585
rs1957937
rs9955360
rs11412296
rs17445108
rs41269079
rs117512405
rs10773176
rs9600019
rs13230646
rs11699829
rs142426306
rs73730598
rs35446936
rs4530278
rs9878436
rs141214782
rs12369950
rs6669563
rs61405042
rs9398196
rs112394943
rs2538745
rs7099229
rs939916
rs131797
rs12412214
rs10805346
rs2555104
rs11426156
rs2977608
rs6007020
All
rs4695407
rs202034370
rs145114957
rs6776756
rs77732866
rs12925933
rs139669835
rs117034449
rs11579626
rs144204502
rs6659669
rs1003322
rs111950327
rs3767952
rs1907702
rs143190905
rs185174247
rs10845387
rs4498805
rs1332941
rs10905255
rs41304832
rs137901416
rs6790988
rs6587577
rs3891167
rs61748181
rs77231040
rs150150565
rs80324517
rs34550383
rs182059586
rs28577594
rs1985369
rs8105767
rs611646
rs11085072
rs45604339
rs869785
rs76219171
rs1291143
rs6751209
rs8102497
rs17803849
rs11557154
rs7772289
rs79977579
rs2293579
rs66731853
rs11117354
rs80116508
rs871134
rs28502153
rs9940099
rs1611236
rs56799554
rs5742915
rs4724
rs2967355
rs59409453
rs2276182
rs75664430
rs76065543
rs35640778
rs7705526
rs2763979
rs9419958
SNP
0.00
0.03
0.06
0.09
Effect size (b)
